# Hyperglycemia during the first 1000 days as a driver of metabolic programming

**DOI:** 10.3389/fendo.2026.1899593

**Published:** 2026-07-22

**Authors:** Marton Schandl, Tibor Ertl, Reka A. Vass

**Affiliations:** 1 Department of Obstetrics and Gynecology, Medical School, University of Pécs, Pécs, Hungary; 2 Clinical Centre, University of Pécs, Pécs, Hungary

**Keywords:** developmental origins of health and disease (DOHaD), epigenetics, fetal programming, gestational diabetes mellitus, hyperglycemia, metabolic syndrome, placental dysfunction

## Abstract

The first 1000 days of life, spanning from conception to the end of the second postnatal year, represent a critical developmental window during which environmental and metabolic exposures exert long-lasting effects on offspring health. Among these exposures, maternal and early-life hyperglycemia have emerged as major determinants of metabolic programming and future cardiometabolic disease risk. Increasing evidence suggests that hyperglycemic exposure during this vulnerable period induces complex alterations in placental function, fetal endocrine adaptation, epigenetic regulation, and postnatal metabolic homeostasis, thereby predisposing offspring to obesity, insulin resistance, type 2 diabetes mellitus, and neurodevelopmental disturbances later in life. This review summarizes current evidence regarding the mechanistic pathways linking hyperglycemia during the first 1000 days to adverse metabolic outcomes. We discuss the role of maternal hyperglycemia in placental dysfunction, oxidative stress, inflammation, and altered nutrient transport, as well as its effects on fetal pancreatic development, adipogenesis, and insulin signaling. Particular emphasis is placed on emerging evidence implicating epigenetic modifications, mitochondrial dysfunction, microbiome alterations, and endocrine dysregulation in developmental programming. We further examine the impact of neonatal and early infant metabolic exposures on growth trajectories, adiposity, neurodevelopment, and long-term cardiometabolic health.

Finally, we highlight current knowledge gaps and potential opportunities for early intervention, including optimized glycemic control during pregnancy, nutritional modulation, breastfeeding promotion, and precision prevention strategies targeting high-risk mother–infant dyads. A deeper understanding of the biological mechanisms underlying hyperglycemia-induced metabolic programming may facilitate the development of preventive approaches aimed at reducing the intergenerational transmission of metabolic disease.

## 1 Introduction

The first 1000 days of life, spanning from conception to approximately two years of age, constitute a critical developmental window characterized by rapid cellular differentiation, organogenesis, and metabolic maturation (1). Increasing evidence derived from the framework of the Developmental Origins of Health and Disease (DOHaD) suggests that environmental and metabolic exposures during this vulnerable period may exert long-term effects on health trajectories and disease susceptibility across the lifespan (2, 3). Among these exposures, maternal and early-life hyperglycemia have emerged as major contributors to adverse metabolic programming and intergenerational transmission of cardiometabolic disease risk.

The global prevalence of hyperglycemia during pregnancy has increased substantially over recent decades, largely driven by rising rates of obesity, sedentary lifestyle, advanced maternal age, and type 2 diabetes mellitus (4). Gestational diabetes mellitus (GDM), the most common form of hyperglycemia in pregnancy, affects approximately 14–16% of pregnancies worldwide, although prevalence varies according to diagnostic criteria and population characteristics (5). In addition to overt diabetes and GDM, milder forms of maternal dysglycemia have also been associated with adverse fetal and neonatal outcomes (6). Maternal hyperglycemia is increasingly recognized not merely as a transient metabolic disturbance, but as a potent developmental signal capable of altering placental physiology, fetal endocrine adaptation, and long-term metabolic homeostasis in offspring.

Hyperglycemic exposure during fetal life induces a complex cascade of biological alterations involving oxidative stress, chronic low-grade inflammation, mitochondrial dysfunction, and abnormal nutrient transport across the placenta (7, 8). These disturbances may contribute to fetal hyperinsulinemia, altered pancreatic β-cell development, excessive adipogenesis, and insulin resistance (9). Furthermore, accumulating evidence suggests that hyperglycemia-induced epigenetic modifications, including DNA methylation, histone modifications, and altered microRNA expression, may mediate persistent changes in gene expression and metabolic phenotype extending into childhood and adulthood (10, 11).

In addition to intrauterine programming, postnatal metabolic exposures during infancy may further influence disease susceptibility. Early infant feeding practices, accelerated postnatal growth, microbiome composition, and neonatal endocrine adaptations interact with prenatal metabolic programming to shape long-term cardiometabolic outcomes (12). Offspring exposed to maternal hyperglycemia demonstrate increased risks of childhood obesity, impaired glucose tolerance, hypertension, neurodevelopmental disturbances, and type 2 diabetes later in life (13, 14). These findings support the concept that metabolic disease may originate, at least in part, during early developmental periods.

Despite growing recognition of the impact of hyperglycemia during the first 1000 days, significant gaps remain regarding the precise molecular mechanisms underlying developmental programming and the identification of effective preventive strategies. A comprehensive understanding of the placental, endocrine, epigenetic, and microbial pathways involved may facilitate the development of targeted interventions aimed at interrupting the intergenerational cycle of metabolic disease.

This review summarizes current evidence regarding the role of hyperglycemia during the first 1000 days as a driver of metabolic programming. We discuss the effects of maternal hyperglycemia on placental function, fetal metabolic adaptation, epigenetic regulation, neonatal endocrine programming, microbiome alterations, and long-term offspring outcomes, while also highlighting emerging therapeutic and preventive opportunities.

## 2 Maternal hyperglycemia and placental dysfunction

The placenta plays a pivotal role in regulating fetal growth and development by coordinating nutrient transport, endocrine signaling, gas exchange, and immunological communication between the maternal and fetal compartments. In pregnancies complicated by hyperglycemia, the placenta functions not merely as a passive interface but as a dynamic metabolic sensor capable of mediating developmental programming and long-term disease susceptibility in offspring (7, 8). Maternal hyperglycemia induces substantial structural, molecular, and functional alterations within placental tissue, thereby contributing to an adverse intrauterine environment associated with future cardiometabolic dysfunction.

Glucose represents the primary energy substrate for fetal development and is transported across the placenta predominantly via facilitated diffusion mediated by glucose transporter proteins, particularly glucose transporter 1 (GLUT1). Hyperglycemic pregnancies have been associated with increased placental GLUT1 expression and enhanced transplacental glucose transfer, resulting in excessive fetal glucose exposure and compensatory fetal hyperinsulinemia (6, 9, 15). Since maternal insulin does not cross the placental barrier, fetal insulin secretion constitutes a direct adaptive response to elevated intrauterine glucose concentrations. Chronic fetal hyperinsulinemia subsequently promotes enhanced adipogenesis, accelerated somatic growth, altered insulin signaling, and increased fat deposition, all of which may predispose offspring to obesity and insulin resistance later in life (9, 16).

In addition to dysregulated glucose transport, maternal hyperglycemia contributes significantly to placental oxidative stress and chronic inflammation. Excess glucose availability stimulates mitochondrial overproduction of reactive oxygen species (ROS), resulting in lipid peroxidation, DNA damage, protein oxidation, and impaired intracellular signaling pathways (8, 17). Oxidative stress is further exacerbated by activation of inflammatory cascades characterized by elevated placental expression of pro-inflammatory cytokines, including tumor necrosis factor-α (TNF-α), interleukin-6 (IL-6), and interleukin-1β (IL-1β) (7, 18). These inflammatory mediators impair insulin signaling pathways and endothelial function while disrupting trophoblast differentiation and placental vascular development. Consequently, placental inflammation and oxidative stress may alter nutrient sensing and transport mechanisms, thereby amplifying fetal metabolic dysregulation.

Mitochondrial dysfunction has emerged as another central mechanism underlying placental pathology in hyperglycemic pregnancies. Experimental and clinical studies have demonstrated that hyperglycemia impairs mitochondrial biogenesis, disrupts oxidative phosphorylation, and alters mitochondrial dynamics within trophoblastic cells (17, 19). These abnormalities compromise placental energy metabolism and may contribute to defective angiogenesis, impaired oxygen delivery, and increased placental apoptosis. Furthermore, mitochondrial dysfunction has been implicated in accelerated placental cellular senescence, potentially exacerbating placental insufficiency and adverse fetal outcomes (19).

Altered placental lipid metabolism also contributes substantially to fetal metabolic programming. Maternal hyperglycemia is frequently accompanied by dyslipidemia, hypertriglyceridemia, and elevated circulating free fatty acids, all of which enhance placental lipid uptake and storage (7, 20). Excessive lipid accumulation within placental tissue promotes lipotoxicity, inflammation, endoplasmic reticulum stress, and further oxidative injury. These metabolic disturbances may influence fetal adipocyte differentiation and hepatic lipid metabolism, thereby increasing susceptibility to childhood obesity and metabolic syndrome (20, 21).

Emerging evidence additionally suggests that maternal hyperglycemia disrupts placental endocrine function. The placenta synthesizes numerous hormones and bioactive molecules, including human placental lactogen, leptin, adiponectin, corticotropin-releasing hormone, and insulin-like growth factors, which collectively regulate fetal growth and maternal metabolic adaptation. Hyperglycemic conditions may alter the secretion and signaling of these hormones, potentially affecting fetal appetite regulation, hypothalamic development, and long-term endocrine homeostasis (14, 22). Moreover, placental exposure to hyperglycemia has been associated with epigenetic modifications involving DNA methylation, histone modification, and altered microRNA expression, resulting in persistent changes in genes regulating inflammation, metabolism, and insulin sensitivity (10, 11, 23).

**Figure 1:**
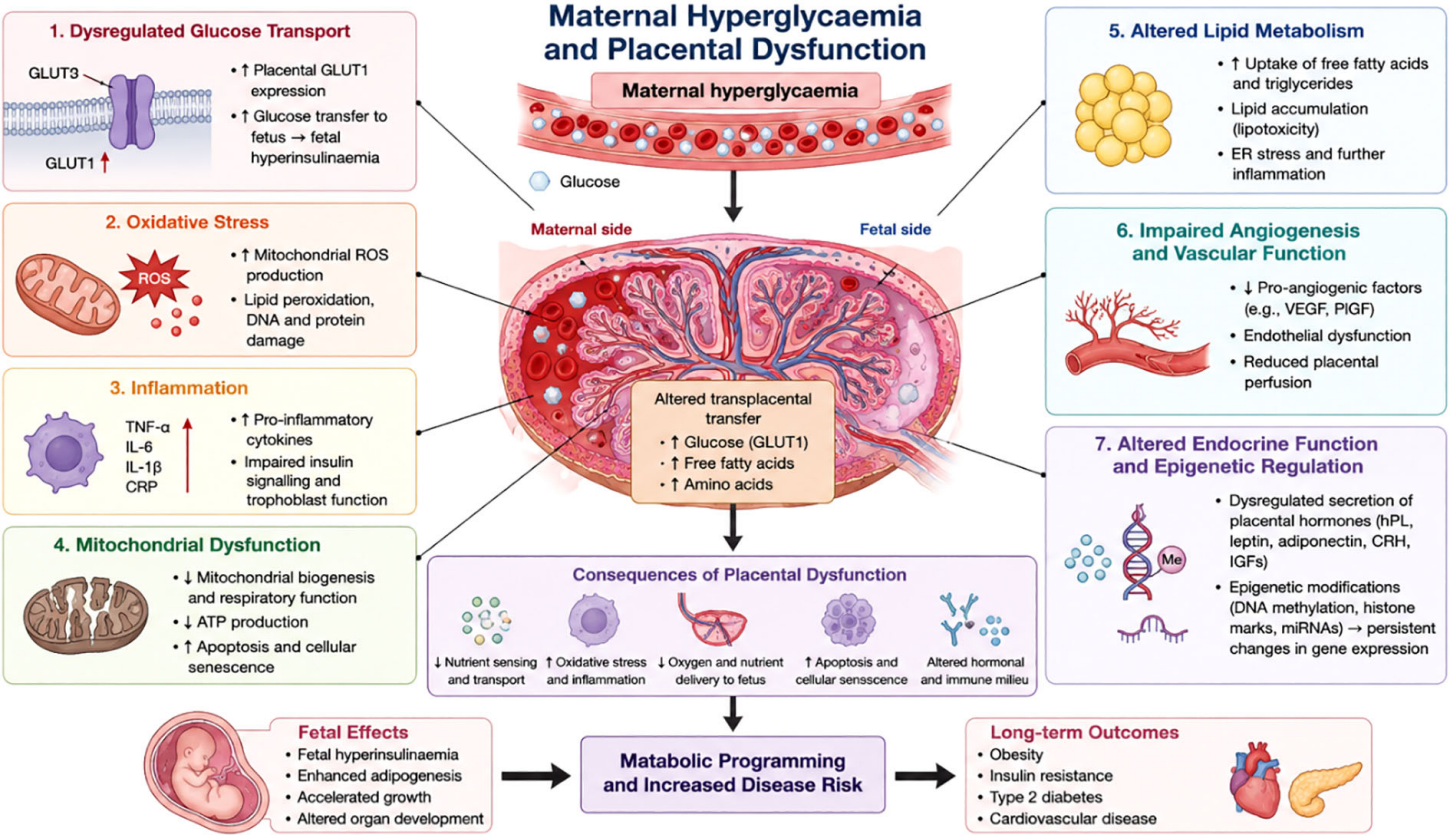
Maternal hyperglycemia-induced placental dysfunction as a central mediator of fetal metabolic programming. Maternal hyperglycemia induces multiple interrelated structural and functional alterations within the placenta, including dysregulated glucose transport, oxidative stress, chronic inflammation, mitochondrial dysfunction, impaired angiogenesis, altered lipid metabolism, and disrupted endocrine signaling. Increased placental GLUT1 expression enhances transplacental glucose transfer, resulting in excessive fetal glucose exposure and compensatory fetal hyperinsulinemia. Hyperglycemic conditions further promote reactive oxygen species (ROS) generation, inflammatory cytokine production, endothelial dysfunction, and trophoblastic mitochondrial impairment, thereby compromising placental nutrient sensing and vascular homeostasis. Concurrent alterations in placental lipid handling and endocrine function may additionally contribute to epigenetic modifications and persistent changes in fetal metabolic pathways. Collectively, these placental disturbances create an adverse intrauterine environment that promotes fetal adipogenesis, altered organ development, and long-term susceptibility to obesity, insulin resistance, type 2 diabetes mellitus, and cardiometabolic disease. GLUT1, glucose transporter 1; ROS, reactive oxygen species; TNF-α, tumor necrosis factor-α; IL-6, interleukin-6; IL-1β, interleukin-1β; VEGF, vascular endothelial growth factor; PlGF, placental growth factor; hPL, human placental lactogen; CRH, corticotropin-releasing hormone; IGF, insulin-like growth factor.

Collectively, these findings support the concept that the placenta serves as a central mediator linking maternal hyperglycemia to fetal metabolic programming. Through complex interactions involving altered nutrient transport, oxidative stress, inflammation, mitochondrial dysfunction, lipid dysregulation, and endocrine signaling, placental dysfunction may establish the biological foundation for long-term cardiometabolic disease susceptibility in offspring (**Figure 1**).

## 3 Fetal metabolic adaptations to hyperglycemic exposure

Fetal development is characterized by remarkable metabolic plasticity that enables adaptation to changes in the intrauterine environment. In pregnancies complicated by maternal hyperglycemia, the fetus undergoes a series of adaptive metabolic responses aimed at maintaining energy homeostasis and supporting growth under conditions of excessive nutrient exposure. Although these adaptations may initially promote fetal survival and growth, persistent exposure to hyperglycemia can induce long-term alterations in endocrine, metabolic, and cellular pathways that increase susceptibility to obesity, insulin resistance, and cardiometabolic disease later in life (9, 16).

One of the earliest and most prominent fetal responses to maternal hyperglycemia is the development of fetal hyperinsulinemia. Since glucose readily crosses the placenta while maternal insulin does not, elevated maternal glucose concentrations stimulate excessive fetal pancreatic insulin secretion (6, 9). Insulin acts as a potent fetal growth factor by promoting glucose uptake, glycogen synthesis, lipogenesis, and protein synthesis within fetal tissues (24). Chronic fetal hyperinsulinemia therefore contributes substantially to accelerated fetal growth and macrosomia, particularly through enhanced adipose tissue deposition and increased hepatic glycogen storage (25). This concept forms the basis of the classical Pedersen hypothesis, which proposes that maternal hyperglycemia drives fetal overgrowth via compensatory fetal insulin secretion (26).

Persistent exposure to hyperglycemia may also alter pancreatic β-cell development and function. Experimental studies suggest that chronic fetal glucose overstimulation initially induces β-cell hyperplasia and increased insulin production; however, prolonged metabolic stress may subsequently impair β-cell maturation, reduce insulin secretory capacity, and predispose offspring to pancreatic dysfunction later in life (27). Altered β-cell programming during fetal development may contribute to impaired glucose tolerance and increased risk of type 2 diabetes mellitus in adulthood (13, 27).

In addition to pancreatic alterations, fetal hyperglycemic exposure affects adipose tissue development and adipocyte differentiation. Hyperinsulinemia stimulates adipogenesis through activation of lipogenic pathways and increased expression of adipogenic transcription factors, including peroxisome proliferator-activated receptor gamma (PPAR-γ) and sterol regulatory element-binding proteins (SREBPs) (28). Offspring exposed to maternal hyperglycemia often exhibit increased neonatal fat mass and altered body composition independent of birth weight, suggesting that intrauterine metabolic programming may influence adiposity trajectories from early life onward (29). Furthermore, excessive fetal lipid exposure may impair adipocyte metabolic flexibility and contribute to chronic low-grade inflammation and insulin resistance in later childhood.

Skeletal muscle and hepatic metabolism are also profoundly influenced by fetal exposure to maternal hyperglycemia. Skeletal muscle represents a major site of insulin-mediated glucose disposal, and experimental evidence indicates that intrauterine hyperglycemia may impair insulin receptor signaling and glucose transporter expression within fetal muscle tissue (30). Concurrently, altered hepatic glucose metabolism, increased gluconeogenic activity, and enhanced lipid accumulation may predispose offspring to hepatic insulin resistance and non-alcoholic fatty liver disease later in life (31). These metabolic disturbances collectively contribute to long-term impairment of glucose homeostasis.

Hyperglycemic exposure during fetal life may additionally disrupt hypothalamic development and appetite regulation. The fetal hypothalamus undergoes critical maturation during late gestation and is highly sensitive to hormonal and nutritional signals. Excessive exposure to glucose, insulin, leptin, and inflammatory mediators may alter neuronal development within appetite-regulating centers, thereby influencing satiety signaling, feeding behavior, and long-term energy balance (32). Such alterations may contribute to increased susceptibility to obesity and metabolic syndrome during childhood and adulthood.

Emerging evidence further suggests that fetal adaptations to maternal hyperglycemia involve alterations in mitochondrial function and cellular energy metabolism. Hyperglycemic conditions may impair mitochondrial oxidative phosphorylation and increase oxidative stress within fetal tissues, leading to reduced metabolic efficiency and increased cellular vulnerability (17, 33). Mitochondrial dysfunction during fetal development has been implicated in impaired insulin sensitivity, altered muscle metabolism, and increased cardiometabolic risk later in life (**Table 1**).

**Table 1:** Mechanisms linking maternal hyperglycemia to offspring metabolic programming during the first 1000 days.

Mechanism	Biological pathway	Placental/Fetal effect	Long-term offspring outcome	References
Oxidative stress	ROS overproduction	Placental dysfunction	Insulin resistance	(8, 17)
Inflammation	TNF-α, IL-6 activation	Endothelial dysfunction	Obesity/metabolic syndrome	(7, 18)
Hyperinsulinemia	β-cell overstimulation	Increased adipogenesis	Type 2 diabetes	(9, 24)
Epigenetic modification	DNA methylation	Altered gene expression	Lifelong metabolic risk	(10, 34)
Mitochondrial dysfunction	Impaired oxidative phosphorylation	Reduced energy metabolism	Cardiometabolic disease	(17, 33)

Collectively, these findings support the concept that maternal hyperglycemia acts as a potent developmental programming signal capable of reshaping fetal metabolic pathways. Although fetal adaptations initially represent compensatory responses to excessive nutrient exposure, persistent metabolic alterations involving pancreatic function, adipogenesis, insulin signaling, hepatic metabolism, hypothalamic regulation, and mitochondrial activity may establish the biological foundation for long-term cardiometabolic disease susceptibility.

## 4 Epigenetic mechanisms of metabolic programming

Increasing evidence suggests that epigenetic modifications represent one of the principal mechanisms linking maternal hyperglycemia during the first 1000 days to long-term metabolic disease susceptibility in offspring. Epigenetic regulation refers to heritable yet potentially reversible alterations in gene expression that occur without changes in the underlying DNA sequence. These mechanisms include DNA methylation, histone modifications, chromatin remodeling, and non-coding RNA-mediated regulation, all of which play essential roles in fetal development and metabolic adaptation (10, 11). Because epigenetic processes are particularly dynamic during early embryonic and fetal life, exposure to adverse intrauterine environments such as hyperglycemia may induce persistent molecular changes that influence metabolic phenotype throughout the lifespan.

Among the various epigenetic mechanisms, DNA methylation has been most extensively investigated in the context of maternal hyperglycemia. DNA methylation typically occurs at cytosine-phosphate-guanine (CpG) dinucleotides and may suppress or enhance gene transcription depending on genomic location. Pregnancies complicated by gestational diabetes mellitus (GDM) have been associated with altered methylation patterns in placental tissue, umbilical cord blood, and fetal metabolic tissues (10, 11, 23). Several genes involved in glucose metabolism, insulin signaling, adipogenesis, and inflammation demonstrate differential methylation following intrauterine hyperglycemic exposure, including leptin (LEP), adiponectin (ADIPOQ), insulin-like growth factor 2 (IGF2), peroxisome proliferator-activated receptor gamma coactivator-1 alpha (PGC-1α), and pancreatic and duodenal homeobox 1 (PDX1) (23, 34, 35). These epigenetic alterations may contribute to impaired insulin sensitivity, altered adipocyte differentiation, and dysregulated energy homeostasis later in life.

Hyperglycemia-induced epigenetic modifications may also affect pancreatic β-cell development and function. Experimental studies suggest that chronic intrauterine glucose excess alters the epigenetic regulation of genes critical for β-cell maturation and insulin secretion, thereby impairing pancreatic function in offspring (35, 36). Reduced expression of PDX1, a key transcription factor involved in pancreatic development, has been associated with increased DNA methylation and histone deacetylation in animal models exposed to adverse intrauterine metabolic conditions (36). Such changes may predispose offspring to impaired glucose tolerance and type 2 diabetes mellitus during adulthood.

Histone modifications represent another important epigenetic mechanism implicated in fetal metabolic programming. Histone acetylation and methylation regulate chromatin accessibility and transcriptional activity, thereby influencing cellular differentiation and metabolic signaling pathways. Maternal hyperglycemia may alter histone-modifying enzymes and chromatin structure within placental and fetal tissues, leading to persistent changes in inflammatory, oxidative stress, and metabolic gene expression (37). Experimental evidence suggests that altered histone acetylation patterns may contribute to mitochondrial dysfunction, adipocyte hypertrophy, and insulin resistance in offspring exposed to maternal diabetes (38).

Non-coding RNAs, particularly microRNAs (miRNAs), have also emerged as key regulators of developmental programming. miRNAs are small non-coding RNA molecules that regulate post-transcriptional gene expression and participate in numerous biological processes including cell proliferation, insulin signaling, lipid metabolism, and inflammation. Hyperglycemic pregnancies have been associated with altered placental and fetal miRNA expression profiles, including dysregulation of miR-29, miR-103, miR-126, and miR-143, all of which are involved in metabolic and vascular pathways (17, 39). Abnormal miRNA signaling may impair trophoblast function, endothelial homeostasis, mitochondrial activity, and fetal insulin sensitivity, thereby contributing to long-term cardiometabolic dysfunction.

Importantly, epigenetic alterations induced during fetal life may persist beyond birth and potentially exert transgenerational effects. Several epidemiological and experimental studies suggest that maternal metabolic disturbances can influence not only immediate offspring health but also disease susceptibility in subsequent generations (40). This phenomenon may partially explain the intergenerational transmission of obesity, insulin resistance, and type 2 diabetes mellitus observed in populations with high prevalence of maternal hyperglycemia. Furthermore, epigenetic programming may interact with postnatal environmental exposures, including infant nutrition, growth patterns, and lifestyle factors, thereby modulating long-term metabolic trajectories.

Recent advances in epigenomics and multi-omics technologies have substantially expanded understanding of the molecular pathways underlying developmental programming. High-throughput analyses of DNA methylation, histone modifications, transcriptomics, and metabolomics have identified numerous candidate pathways linking maternal hyperglycemia to offspring metabolic disease (41). However, many questions remain regarding tissue specificity, temporal stability, sex-dependent effects, and reversibility of epigenetic alterations induced during early life (**Table 2**).

**Table 2:** Epigenetic modifications associated with maternal hyperglycemia.

Epigenetic target/pathway	Epigenetic alteration	Tissue or model studied	Biological consequence	Potential long-term outcome	References
LEP (Leptin)	Differential DNA methylation	Placenta, cord blood	Altered leptin expression and appetite regulation	Increased adiposity and obesity risk	(23, 34)
ADIPOQ (Adiponectin)	DNA methylation changes	Placental and fetal tissues	Impaired adiponectin signaling and insulin sensitivity	Insulin resistance and metabolic syndrome	(35)
IGF2/H19 locus	Aberrant methylation and imprinting alterations	Placenta, fetal tissues	Dysregulated fetal growth signaling	Macrosomia and future cardiometabolic risk	(38)
PDX1	DNA hypermethylation and histone deacetylation	Pancreatic β-cells (animal models)	Impaired β-cell maturation and insulin secretion	Type 2 diabetes mellitus susceptibility	(36)
PGC-1α	Promoter methylation	Skeletal muscle and metabolic tissues	Mitochondrial dysfunction and impaired oxidative metabolism	Reduced insulin sensitivity	(37)
Inflammatory cytokine pathways (TNF-α, IL-6)	Histone modification and altered chromatin accessibility	Placental tissue	Enhanced inflammatory signaling	Chronic low-grade inflammation and obesity	(18, 37)
miR-29 family	Altered microRNA expression	Placenta and fetal circulation	Dysregulated insulin signaling and glucose metabolism	Increased diabetes risk	(39)
miR-103/107	Upregulated microRNA expression	Experimental metabolic models	Impaired insulin receptor signaling	Early insulin resistance	(39)
miR-126	Reduced expression	Placental endothelial tissue	Endothelial dysfunction and impaired angiogenesis	Cardiovascular and metabolic dysfunction	(39)
miR-143	Dysregulated expression	Placental trophoblasts	Altered mitochondrial metabolism and adipocyte differentiation	Obesity and metabolic dysregulation	(17, 39)
Global placental methylation patterns	Widespread epigenomic remodeling	Placenta and cord blood	Persistent alterations in metabolic gene expression	Lifelong cardiometabolic susceptibility	(10, 11, 41)
Histone acetylation pathways	Altered histone acetyltransferase/deacetylase activity	Experimental fetal tissues	Dysregulated chromatin structure and metabolic gene transcription	Impaired glucose homeostasis	(37, 38)
Mitochondrial regulatory genes	Epigenetic dysregulation	Placental and fetal metabolic tissues	Impaired oxidative phosphorylation and increased ROS production	Metabolic inflexibility and insulin resistance	(17, 33)

ADIPOQ, adiponectin gene; CpG, cytosine-phosphate-guanine; DNA, deoxyribonucleic acid; IGF2, insulin-like growth factor 2; IL-6, interleukin-6; LEP, leptin gene; miRNA (miR), microRNA; PDX1, pancreatic and duodenal homeobox 1; PGC-1α, peroxisome proliferator-activated receptor gamma coactivator-1 alpha; ROS, reactive oxygen species; TNF-α, tumor necrosis factor-alpha.

**Figure 2:**
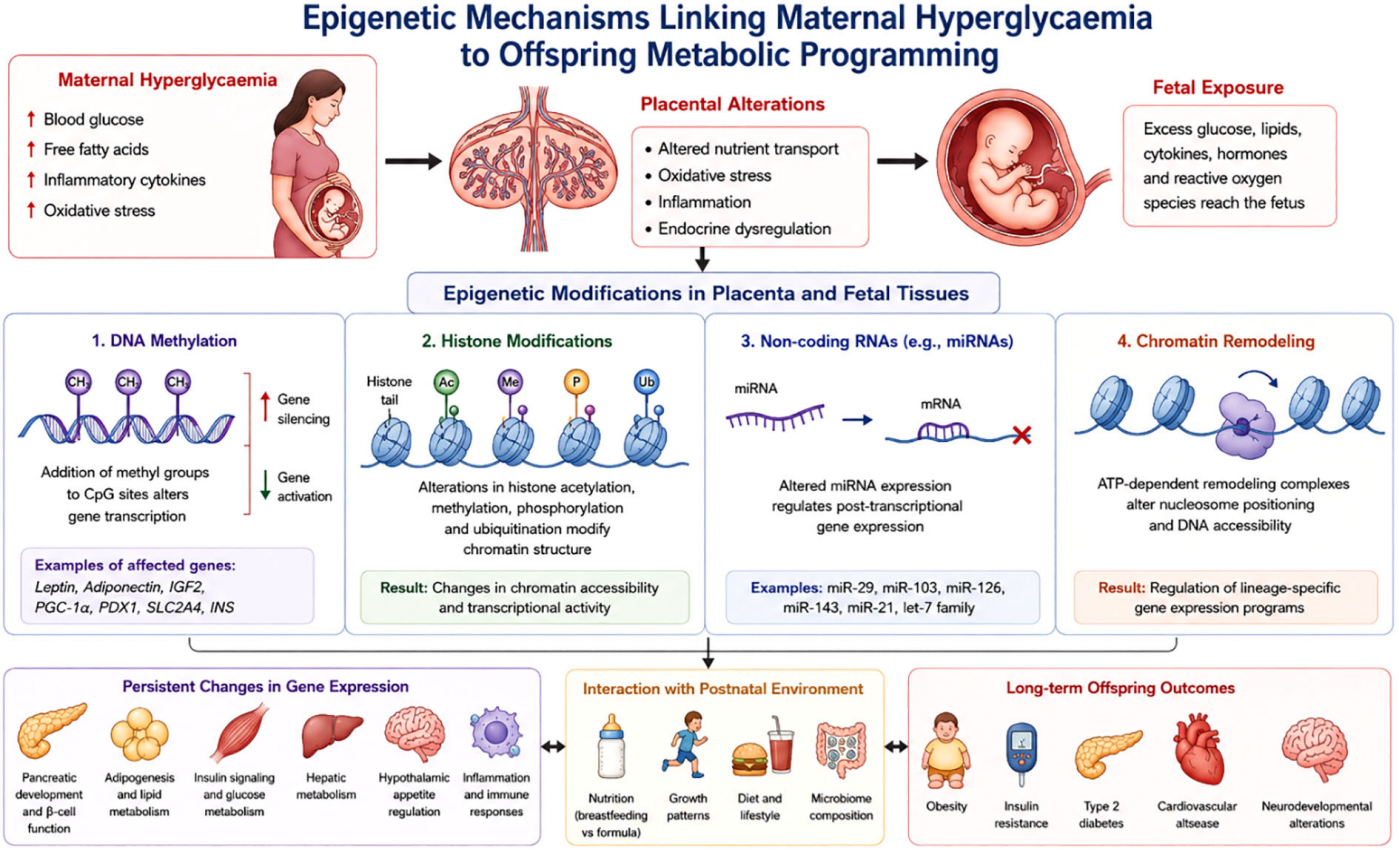
Epigenetic mechanisms linking maternal hyperglycemia to offspring metabolic programming. Maternal hyperglycemia induces multiple placental and fetal alterations that promote epigenetic modifications during critical developmental windows. Excess intrauterine exposure to glucose, lipids, inflammatory cytokines, and oxidative stress mediators may alter fetal gene expression through several interconnected epigenetic mechanisms, including DNA methylation, histone modifications, non-coding RNA dysregulation, and chromatin remodeling. These molecular alterations affect genes involved in pancreatic β-cell development, adipogenesis, insulin signaling, hepatic glucose metabolism, hypothalamic appetite regulation, mitochondrial function, and inflammatory pathways. Persistent epigenetic changes established during fetal life may interact with postnatal environmental exposures such as nutrition, growth patterns, lifestyle factors, and microbiome composition, thereby contributing to long-term susceptibility to obesity, insulin resistance, type 2 diabetes mellitus, cardiovascular disease, and neurodevelopmental dysfunction. The figure highlights the central role of epigenetic regulation as a mechanistic bridge between maternal metabolic disturbances and lifelong offspring cardiometabolic health.

Collectively, current evidence supports the concept that epigenetic mechanisms serve as critical mediators between maternal hyperglycemia and long-term metabolic programming in offspring. Through persistent alterations in gene regulation affecting pancreatic development, adipogenesis, insulin signaling, mitochondrial function, and inflammatory pathways, epigenetic modifications may constitute the molecular foundation for lifelong cardiometabolic disease susceptibility (**Figure 2**).

## 5 Neonatal endocrine programming

The neonatal period represents a critical transitional phase during which the infant adapts from the intrauterine environment to independent metabolic and endocrine regulation. This period is characterized by rapid hormonal, metabolic, and physiological adjustments that are essential for maintaining glucose homeostasis, thermoregulation, growth, and organ maturation. Exposure to maternal hyperglycemia during fetal life may disrupt these adaptive processes, leading to persistent alterations in endocrine signaling and metabolic regulation that contribute to long-term cardiometabolic disease susceptibility (14, 24).

One of the most immediate endocrine consequences observed in neonates born to mothers with hyperglycemia is neonatal hypoglycemia. Chronic intrauterine glucose excess stimulates fetal pancreatic β-cell hyperplasia and persistent hyperinsulinemia, which may continue after birth despite the sudden interruption of maternal glucose supply (9, 24). Excess insulin secretion during the early neonatal period may impair glucose homeostasis and increase the risk of symptomatic hypoglycemia, particularly among infants of diabetic mothers (42). (43–45).

Maternal hyperglycemia may also influence neonatal leptin and adiponectin signaling, both of which play critical roles in appetite regulation, energy balance, insulin sensitivity, and adipose tissue development. Neonates exposed to hyperglycemic intrauterine environments frequently exhibit elevated circulating leptin concentrations secondary to increased adiposity and altered placental leptin production (46). Dysregulated leptin exposure during early life may impair hypothalamic appetite-regulating pathways and contribute to long-term obesity risk. In contrast, reduced adiponectin concentrations have been associated with impaired insulin sensitivity and increased metabolic dysfunction in offspring exposed to maternal diabetes (47).

The hypothalamic–pituitary–adrenal (HPA) axis also appears particularly sensitive to early hyperglycemic exposure. Experimental and clinical studies suggest that maternal metabolic disturbances may alter fetal and neonatal glucocorticoid signaling, thereby affecting stress responsiveness, neuroendocrine regulation, immune function, and energy metabolism (48). Excess glucocorticoid exposure during critical developmental windows may impair hypothalamic maturation and contribute to long-term alterations in appetite regulation, insulin sensitivity, and cardiovascular function.

Insulin signaling pathways remain central to neonatal endocrine programming. In addition to regulating glucose metabolism, insulin exerts important trophic effects on developing tissues, including the liver, skeletal muscle, adipose tissue, and central nervous system. Persistent neonatal hyperinsulinemia may alter insulin receptor sensitivity and downstream metabolic signaling pathways, thereby promoting adipocyte hypertrophy, impaired glucose utilization, and early insulin resistance (27, 30). These metabolic disturbances may persist into childhood and adulthood, particularly when combined with rapid postnatal weight gain and obesogenic environmental exposures.

Breastfeeding and early infant nutrition further influence neonatal endocrine adaptation and may partially modulate the adverse effects of intrauterine hyperglycemic exposure. Human breast milk contains numerous bioactive compounds, including leptin, adiponectin, insulin, growth factors, and immunomodulatory molecules that contribute to metabolic and endocrine maturation (49). Breastfeeding has been associated with improved glucose metabolism, reduced childhood obesity risk, and enhanced insulin sensitivity among offspring of mothers with gestational diabetes mellitus (50). Conversely, early formula feeding and accelerated postnatal growth may amplify metabolic programming effects and increase cardiometabolic disease susceptibility later in life. However, the available evidence is derived predominantly from observational studies, and therefore causal inferences should be made with caution. Although the overall findings consistently suggest a protective association, the magnitude of the effect varies across studies and may be influenced by residual confounding, selection bias, and differences in maternal socioeconomic status, metabolic health, breastfeeding duration, and complementary infant feeding practices. Consequently, the independent contribution of breastfeeding, relative to other maternal and postnatal lifestyle factors, remains to be fully elucidated (44, 45, 51, 52).

Emerging evidence additionally suggests that neonatal endocrine programming may involve interactions between hormonal regulation, immune development, and the gut microbiome. Early-life endocrine disturbances may influence intestinal permeability, microbial colonization, inflammatory signaling, and metabolic homeostasis, thereby further contributing to developmental programming of obesity and insulin resistance (12, 51). These complex interactions highlight the importance of the neonatal period as a vulnerable yet potentially modifiable stage within the first 1000 days.

Collectively, neonatal endocrine programming represents a critical mechanistic link between intrauterine hyperglycemic exposure and long-term metabolic health. Alterations in insulin secretion, leptin and adiponectin signaling, HPA-axis regulation, and early nutritional adaptation may contribute to persistent endocrine and metabolic dysfunction, thereby increasing susceptibility to obesity, insulin resistance, and cardiometabolic disease across the lifespan.

## 6 Microbiome alterations and metabolic programming

The gut microbiome has emerged as an important mediator of developmental programming during the first 1000 days of life. Increasing evidence suggests that maternal hyperglycemia and gestational diabetes mellitus (GDM) influence both maternal and neonatal microbial communities, thereby affecting immune maturation, intestinal barrier integrity, nutrient metabolism, and long-term metabolic health (12, 51, 53). These alterations represent an additional mechanistic pathway linking adverse intrauterine environments with an increased susceptibility to obesity, insulin resistance, and type 2 diabetes mellitus.

Pregnancies complicated by GDM are associated with reduced microbial diversity and altered relative abundances of bacterial taxa involved in carbohydrate fermentation, bile acid metabolism, and inflammatory regulation (54, 55). Maternal dysbiosis may influence fetal development through changes in circulating microbial metabolites, including short-chain fatty acids (SCFAs), bile acids, and lipopolysaccharides, which modulate placental inflammation, insulin sensitivity, and energy homeostasis (56). Beyond their metabolic functions, these microbial metabolites act as endocrine-like signaling molecules within the microbiota–metabolism axis, influencing glucose homeostasis, immune responses, and epigenetic regulation. Consequently, alterations in maternal microbial composition may contribute to fetal metabolic programming through interactions between microbial, endocrine, and inflammatory pathways (12, 51, 56).

Neonatal microbial colonization is further shaped by maternal metabolic status, mode of delivery, antibiotic exposure, and infant feeding practices. Infants born to mothers with GDM exhibit distinct gut microbial profiles during early life, characterized by altered bacterial diversity and composition that may persist beyond infancy (57). Although the long-term consequences remain under investigation, these microbial alterations have been associated with impaired glucose metabolism, increased adiposity, immune dysregulation, and an elevated risk of later cardiometabolic disease.

Breastfeeding is one of the most important modulators of early-life microbiome development. Human milk provides beneficial microorganisms, human milk oligosaccharides, and numerous bioactive compounds that selectively promote the growth of commensal bacteria, particularly *Bifidobacterium* species, thereby supporting intestinal maturation, immune homeostasis, and metabolic development (12, 49). In offspring exposed to maternal hyperglycemia, breastfeeding may partially restore microbial diversity and metabolic function. However, the available evidence is derived predominantly from observational studies, and the magnitude of these beneficial effects varies among cohorts owing to differences in maternal metabolic status, breastfeeding duration, complementary feeding practices, and other postnatal environmental factors.

Despite rapid advances in microbiome research, important knowledge gaps remain regarding the causal role of microbial alterations in developmental metabolic programming. Most available studies are observational, employ heterogeneous sequencing methodologies, and include relatively small cohorts, limiting direct comparisons and causal inference. Future investigations integrating longitudinal microbiome profiling with metabolomic, epigenomic, and clinical data will be essential to clarify the contribution of the microbiota to hyperglycemia-induced developmental programming and to determine whether microbiome-targeted interventions may reduce the intergenerational transmission of metabolic disease.

## 7 Future perspectives

Despite substantial advances in understanding the developmental origins of metabolic disease, several important questions remain unanswered. Future research should move beyond establishing associations between maternal hyperglycemia and offspring outcomes toward elucidating the molecular mechanisms underlying developmental programming. Integrative multi-omics approaches—including epigenomics, transcriptomics, proteomics, metabolomics, and microbiome profiling—combined with advanced bioinformatics and systems biology are expected to provide a more comprehensive understanding of the complex interactions between placental function, fetal metabolism, endocrine regulation, and postnatal environmental influences (58–60).

Large, prospective longitudinal birth cohorts incorporating standardized diagnostic criteria for gestational diabetes mellitus (GDM), detailed maternal metabolic phenotyping, and long-term follow-up of offspring are needed to identify critical windows of susceptibility and validate biomarkers predictive of future cardiometabolic disease. Future studies should also address current knowledge gaps regarding sex-specific responses, the reversibility of hyperglycemia-induced programming, and the interaction between prenatal metabolic exposures and postnatal lifestyle factors, including nutrition, breastfeeding, physical activity, and the gut microbiome (29, 61).

The translation of mechanistic discoveries into clinical practice represents another important priority. Continuous glucose monitoring, individualized nutritional interventions, optimization of gestational weight gain, breastfeeding promotion, and microbiome-targeted strategies have shown promise in improving maternal metabolic control; however, their long-term effects on offspring health remain incompletely understood. Integrating clinical characteristics with molecular biomarkers may facilitate precision prevention strategies that enable early identification of pregnancies at increased risk for adverse developmental programming and allow timely intervention during the first 1000 days (62, 63).

Although maternal hyperglycemia remains the primary determinant of the intrauterine metabolic environment, increasing evidence suggests that paternal metabolic health may also influence offspring metabolic programming. Paternal obesity, diabetes, and other metabolic disturbances before conception have been associated with alterations in the sperm epigenome, including changes in DNA methylation, histone modifications, and small non-coding RNAs, which may influence early embryonic development and increase susceptibility to obesity, insulin resistance, and impaired glucose metabolism in the offspring (64, 65). While these findings are supported predominantly by experimental models and emerging human studies, they highlight the importance of considering parental metabolic health as a shared determinant of developmental programming and warrant further investigation into the contribution of paternal factors to intergenerational cardiometabolic risk.

Overall, future research should focus on integrating mechanistic insights with translational and clinical studies to develop effective preventive strategies that interrupt the intergenerational transmission of metabolic disease. A deeper understanding of the interplay between placental biology, endocrine signaling, epigenetic regulation, the microbiome, and parental metabolic health may ultimately facilitate precision medicine approaches aimed at improving lifelong metabolic outcomes for future generations.

## 8 Conclusion

Hyperglycemia during the first 1000 days of life represents a major developmental exposure capable of exerting long-lasting effects on offspring metabolic health. Increasing evidence suggests that maternal hyperglycemia functions as a potent developmental programming signal influencing placental physiology, fetal metabolic adaptation, endocrine regulation, and epigenetic remodeling during critical periods of development. Through mechanisms involving oxidative stress, inflammation, mitochondrial dysfunction, altered nutrient transport, and dysregulated hormonal signaling, hyperglycemic exposure may predispose offspring to obesity, insulin resistance, type 2 diabetes mellitus, cardiovascular disease, and neurodevelopmental dysfunction later in life.

The placenta serves as a central mediator of these processes by integrating maternal metabolic signals and regulating fetal nutrient and hormonal exposure. Concurrently, fetal adaptive responses—including hyperinsulinemia, altered adipogenesis, impaired pancreatic β-cell development, and disrupted hypothalamic signaling—may contribute to persistent metabolic alterations that extend into adulthood. Epigenetic modifications further provide a mechanistic basis for the long-term and potentially transgenerational effects of early-life hyperglycemic exposure.

Despite significant advances in understanding developmental metabolic programming, important knowledge gaps remain regarding the precise molecular pathways, sex-specific effects, and reversibility of these alterations. Nevertheless, the first 1000 days also represent a critical window for preventive intervention. Improved maternal glycemic control, optimized nutrition, breastfeeding promotion, and early-life metabolic monitoring may help reduce the intergenerational transmission of cardiometabolic disease and improve long-term offspring health outcomes.
